# Systems Medicine as an Emerging Tool for Cardiovascular Genetics

**DOI:** 10.3389/fcvm.2016.00027

**Published:** 2016-08-30

**Authors:** Tina Haase, Daniela Börnigen, Christian Müller, Tanja Zeller

**Affiliations:** ^1^Clinic for General and Interventional Cardiology, University Heart Center Hamburg, Hamburg, Germany; ^2^Partner Site Hamburg/Kiel/Lübeck, German Center for Cardiovascular Research (DZHK e.V.), Hamburg, Germany

**Keywords:** systems medicine, cardiovascular disease, omics, networks, functional characterization

## Abstract

Cardiovascular disease (CVD) is a major contributor to morbidity and mortality worldwide. However, the pathogenesis of CVD is complex and remains elusive. Within the last years, systems medicine has emerged as a novel tool to study the complex genetic, molecular, and physiological interactions leading to diseases. In this review, we provide an overview about the current approaches for systems medicine in CVD. They include bioinformatical and experimental tools such as cell and animal models, omics technologies, network, and pathway analyses. Additionally, we discuss challenges and current literature examples where systems medicine has been successfully applied for the study of CVD.

## Introduction

**Figure 1:**
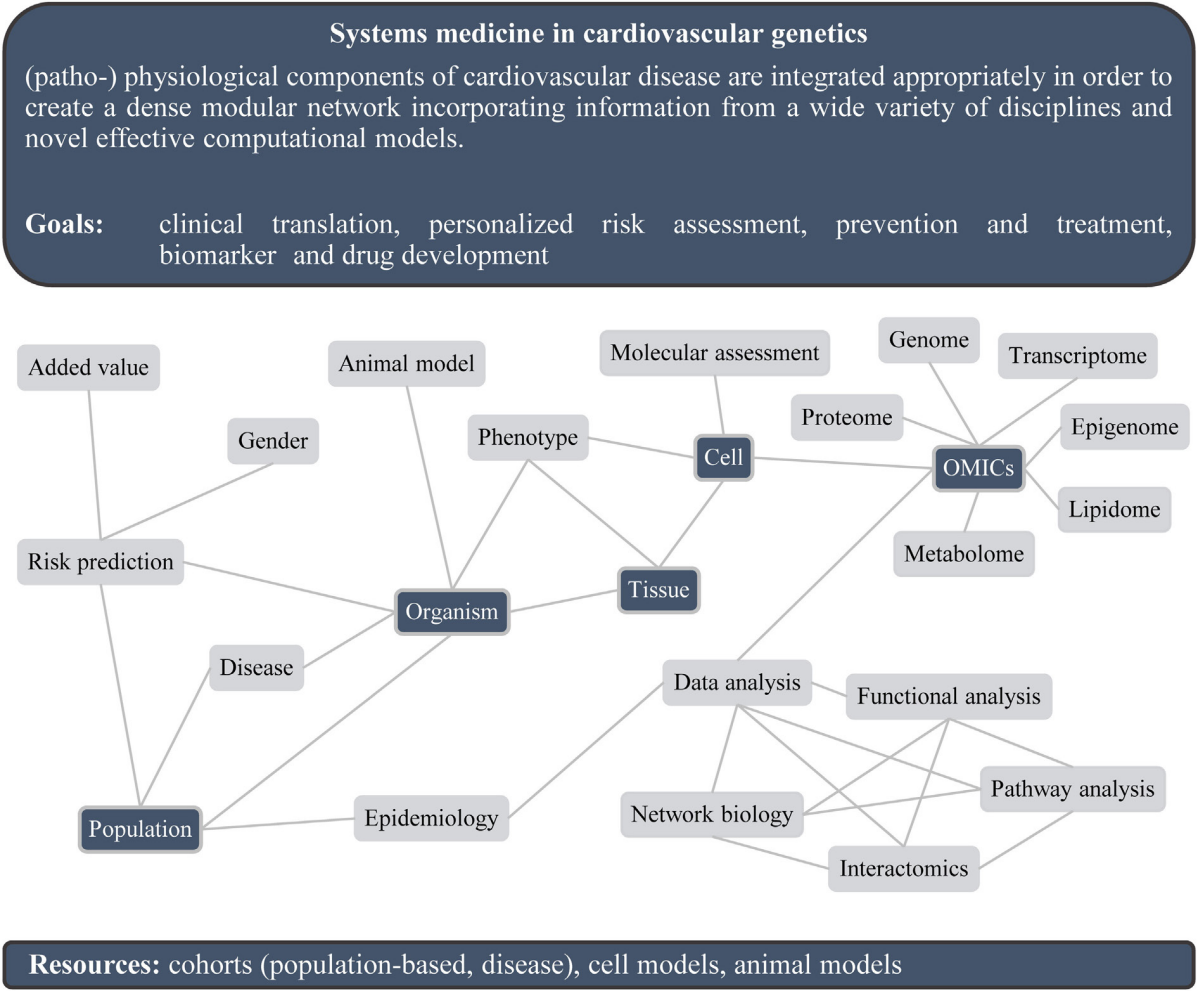
**Systems medicine approaches in cardiovascular genetics**.

Cardiovascular disease (CVD) morbidity and mortality pose a major public health burden worldwide, and the prevalence of CVD is rapidly increasing. CVD is a heritable condition (1) and has a complex and heterogenic etiology involving numerous environmental and genetic factors of disease risk (2). Increasing our understanding of the multifactorial, complex underpinnings of CVD promises to have a global impact on the promotion of health. The invention of arrays and analysis of multiple case–control samples have led to the identification of numerous genetic variants associated with coronary artery disease (CAD) risk. In 2007, the first genome-wide association studies (GWASs) for CAD were published, identifying a locus on chromosome 9p21 (3–5). To date, 56 loci have been identified due to denser genotyping and a higher number of individuals (6). Almost all of them are located in non-coding parts of the genome (6, 7). From these analyses, the locus on chromosome 9p21 is the locus with the highest population-attributable risk. It influences different isoforms of the non-coding RNA ANRIL (8). Besides GWASs, the first exome-wide association study on CAD was published in 2016, not only confirming known variants for CAD such as ANGPTL4 and LPL but also identifying novel variants such as SVEP1 (9). Results from the GWAS area have also led to the development of novel therapeutics, most prominently targeting lipid metabolism through inhibitors for PCSK9 (6). So, despite large success of GWASs (10–12) and sequencing approaches (13, 14) in identifying genetic loci associated with CVD, the underlying pathophysiological mechanisms involve different genotypes and changes in a systems level – for instance, at the transcriptome, proteome, and metabolite levels (Figure 1). To provide a more comprehensive picture, the systematic integration of multidimensional “omics” datasets evolves as the next challenge for the future, including molecular findings of interactions between proteins, metabolites, regulatory RNAs, and DNA as well as knowledge from cell biology, animal experiments, and human phenotypic and clinical data. In this systems approach, all known (patho-)physiological components of CVD are integrated appropriately in order to create a dense modular network incorporating information from various disciplines and novel effective computational models. These approaches will guide the next steps in cardiovascular research, enhance our understanding of disease susceptibility, treatment, and monitoring, and might influence preventive actions (15).

In this review, we discuss state-of-the-art systems medicine approaches in CVD. We provide an overview of existing bioinformatical and experimental methods commonly used in systems medicine and illustrate selected exemplary CVD studies from the current literature.

## Methodology – Omics Approaches

Omics is the study of different biological entities, including genomics, proteomics, transcriptomics, or metabolomics, describing different aspects (components and interactions) of the cell (16).

In systems medicine, such omics data types are integrated to better understand the global relationship among genotype, environment, and phenotype and to reveal the underlying molecular systems and their function (17). Consequently, a more comprehensive picture is taken by the combination of multidimensional omics data covering different levels of knowledge about the cell, genome, or environment.

Subsequent systems medicine approaches, e.g., network and pathway analysis, reveal novel insight into the disease or condition of interest. Here, the relationship among genes, proteins, transcripts, or other biological entities is analyzed and interpreted on a genome-wide scale (e.g., network) or a more detailed scale (e.g., pathway, sub-network).

In the following, we first review selected omics data types and methods for data integration and subsequent data analysis. Table 1 provides additional information along with selected resources (databases, tools) for each presented data type.

**Table 1:** **Tools and resources for omics data**.

Name	Description	Webpage	Reference
**Genomics**			
Ensembl	System for genome annotation, analysis, storage, and dissemination designed to facilitate the access of genomic annotation from chordates and key model organisms	http://www.ensembl.org	(18)
1000 Genomes project	The goal of the 1000 genomes project was to find most genetic variants with frequencies of at least 1% in the populations studied	http://www.1000genomes.org	(19)
UCSC Genome browser	Web tool for rapid and reliable display of any requested portion of the genome at any scale, together with several dozen aligned annotation tracks	https://genome.ucsc.edu	(20)
**Transcriptomics**			
GEO gene expression omnibus	Public functional genomics data repository supporting MIAME-compliant data submissions. Tools are provided to help users query and download experiments and curated gene expression profiles	http://www.ncbi.nlm.nih.gov/geo	(21)
ArrayExpress	Archive of functional genomics data stores data from high-throughput functional genomics experiments	https://www.ebi.ac.uk/arrayexpress	(21)
Expression atlas	Provides information on gene expression patterns under different biological conditions. Gene expression data are re-analyzed in-house to detect genes showing interesting baseline and differential expression patterns	https://www.ebi.ac.uk/gxa	(22)
GXD the mouse gene expression database	Collects and integrates the gene expression information in MGI, focusing on endogenous gene expression during mouse development	http://www.informatics.jax.org/expression.shtml	(23)
**Proteomics**			
PRIDE – PRoteomics IDEntifications	Data repository for proteomics data, including protein and peptide identifications, posttranslational modifications, and supporting spectral evidence	https://www.ebi.ac.uk/pride/archive	(24)
ProteomicsDB	Database for the identification of the human proteome	https://www.proteomicsdb.org	(25)
PeptideAtlas	Compendium of peptides identified in a large set of tandem mass spectrometry proteomics experiments	http://www.peptideatlas.org	(26)
NIST libraries of peptide tandem mass spectra	Comprehensive, annotated mass spectral reference collections from various organisms and proteins	http://peptide.nist.gov	
COPaKB	Proteome biology platform specifically for cardiovascular research	http://www.heartproteome.org	(27)
MitoCarta	Inventory of human and mouse genes encoding proteins with mitochondrial localization	http://www.broadinstitute.org/scientific-community/science/programs/metabolic-disease-program/publications/mitocarta/mitocarta-in-0	(28)
**Metabolomics**			
HMDB – the human metabolome database	Database containing detailed information about small molecule metabolites in human, containing chemical data, clinical data, and molecular biology/biochemistry data	http://www.hmdb.ca	(29)
MetaboLights	Database for metabolomics experiments and derived information. The database is cross-species, cross-technique and covers metabolite structures and their reference spectra, their biological roles, locations and concentrations, and experimental data from metabolic experiments	http://www.ebi.ac.uk/metabolights	(30)
BiGG models	Knowledgebase of genome-scale metabolic network reconstructions, integrating multiple published genome-scale metabolic networks into a single database	http://bigg.ucsd.edu	(31)
MetaCyc	Database of non-redundant, experimentally elucidated metabolic pathways. It is curated from the scientific experimental literature and contains pathways involved in both primary and secondary metabolism, as well as associated compounds, enzymes, and genes	http://metacyc.org	(32)
ConceptMetab	Compound set network tool	http://conceptmetab.med.umich.edu/	(33)
MetDisease	Metabolic network app for Cytoscape	http://metdisease.ncibi.org/	(34)
**Interactomics**			
IntAct – molecular interaction database	Database system and analysis tools for molecular interaction data derived from literature curation or direct user submissions	http://www.ebi.ac.uk/intact	(35)
BioGRID – biological general repository for interaction datasets	Interaction repository with data compiled through comprehensive curation, containing protein and genetic interactions, chemical associations and posttranslational modifications	http://thebiogrid.org	(36)
STRING – protein–protein interaction networks	Database of known and predicted protein–protein interactions, including direct (physical) and indirect (functional) associations	http://string-db.org	(37)

### Genomics

Genomic studies investigate the sequence, structure, and function of the entire genome in a cell. DNA sequencing and assembling is one of the most prominent techniques in genomics. Established techniques and methods involve Shotgun sequencing (e.g., Sanger method), Next-generation sequencing (NGS), and Illumina sequencing. They mainly differ in the length of sequence reads (number of base pairs), coverage, sequencing/processing time, and costs. Whole-genome sequencing analyzes all coding and non-coding regions of the genome. While it has the highest coverage producing large amount of data, it is so far the most expensive method. By contrast, whole exome sequencing covers just the coding sequences of all genes at a high coverage but with cheaper costs. Other techniques include the identification of common single nucleotide polymorphisms (SNPs) by GWASs and CpG methylation sites by epigenome-wide association studies (EWASs), both covering the whole genome (6).

Subsequently, sequenced DNA fragments are assembled and annotated using reference genomes [e.g., RefSeq (38), Genome Reference Consortium]. Such obtained DNA sequences can then be used to study certain mutations, including genetic variants (SNPs), copy number variations (CNVs), and deletions or insertions of gene fragments (39).

### Transciptomics

The transcriptome can be defined as “*the complete set of transcripts in a cell, and their quantity, for a specific developmental stage or physiological condition*” (40). Often, it is used to determine expression patterns of transcripts and how they change under certain conditions, such as disease status and drug treatment. A transcriptome analysis aims, depending on the technique in use, to catalog and annotate RNA, including coding and non-coding transcripts, to query gene structures, and to support constructing and mapping interaction networks.

The most popular technique in transcriptomics analysis is the use of microarrays. They allow us to study tens of thousands of transcripts (RNA) on a genome-wide scale with different conditions in parallel (e.g., disease and healthy). Additionally, they have an extensive coverage, high-throughput applicability, uncomplicated data analysis, and are relatively inexpensive (41). However, microarrays still suffer from several technical limitations (42, 43). They are limited by the amount of RNA required, the dynamic range, the semi-quantitative approach, and the detection of predefined transcripts (41). These limitations have been widely solved by a new technique, RNA-Sequencing (RNA-Seq), which sequences all transcripts in a sample multiple times, obtaining a high-resolution and high-quality genome-wide transcriptome scan (40). RNA-Seq provides absolute quantification of transcripts and includes splice variants, unknown RNAs, and RNAs too short to be captured by microarrays. However, RNA-Seq is a more expensive technology than microarrays, while it requires large data storage and powerful computation resources. Additionally, analysis of RNA-Seq data involves complex bioinformatical approaches (41, 44).

After performing such genome-wide transcript scans, a well-established method for verifying individual promising targets is the use of a quantitative real-time polymerase chain reaction (qPCR). In contrary to microarrays or a genome-wide RNA-Seq analysis, it monitors the amplification of a single target DNA molecule.

In the research of CVD, transcriptomics approaches have already led to the discovery of novel biomarkers, e.g., GDF15 for acute coronary syndromes, angina pectoris, and heart failure (45–47), as well as several circulating microRNAs for coronary heart disease and myocardial infarction (MI) (48–50).

### Proteomics

Proteins have individual interconnected properties that contribute to the phenotype of a cell. Together, all proteins form a complex and dynamic system, the proteome, comprising all interconnected and dynamic properties of the proteins, including their abundance, isoform expression, subcellular localization, interactions, turnover rate, and posttranslational modifications (PTMs) (51).

Proteome studies are still rare in relation to CVD, due to complex methodology involved. A comprehensive review on proteomics in combination with systems biology approaches in CVD is given by Langley et al. (52). In proteomic studies, it is recommended to use plasma rather than serum due to its more stable protein suspension. To avoid masking of low-abundance proteins by high-abundance proteins, such as albumin and immunoglobulins, immunodepletion techniques or protein enrichment tools can be used. Even though protein enrichment tools allow to reduce the dynamic range of protein concentrations, they maintain representatives of all proteins. For initial protein separation, two dimensional (2-D) gel electrophoresis is well established. The introduction of differential in-gel electrophoresis (DIGE) allows separation of two sets of protein mixtures by pre-labeling with fluorescent dyes and gives higher reproducibility than 2-D gels. After separation, protein spots are picked and digested with proteolytic enzymes and subsequently analyzed by tandem mass spectrometry (MS/MS) (53). Gel-free shotgun proteomics include stable isotope labeling with amino acids in cell culture (SILAC), which allows higher protein resolution but is only operable in cell culture experiments and makes quantification more difficult (52).

Although recent advances in mass spectrometry (MS)-based proteomics have resulted in a quantitative system-wide analysis of the proteome, including PTMs, protein–protein interactions (PPIs), and cellular localization, researchers see a next-generation proteomics integrating novel approaches to gain further insight into the proteome by improving sensitivity, robustness, and high-throughput of MS-based proteomics. This allows us to discover novel disease-related biomarkers and screen molecular targets of drugs (54).

### Metabolomics

Metabolites are chemical entities transformed during metabolism that can serve as signatures of biochemical activity. Most metabolites are lipids (phospholipids, glycerophospholipids, and sphingolipids), acylcarnitines, amino acids, biogenic amines, hormones, bile acids, or fatty acids. They are detected quantitatively from body fluids (e.g., serum) or tissues and are measured by nuclear magnetic resonance (NMR) spectroscopy and MS. The following factors should be considered when choosing a method: cost-effectiveness, coverage of metabolic content, accuracy, and throughput. NMR is a quantitative, non-destructive technique, allowing the detection of a wide range of diverse metabolites simultaneously, while sample storage and preparation are very simple. By contrast, MS coupled with ultra-performance liquid chromatography (UPLC-MS) has better resolution power and higher sensitivity than NMR. However, the data quality highly depends on the sample quality. Mainly, two different chromatographic techniques are used for the detection of metabolites: hydrophilic interaction liquid chromatography (HILIC) and reversed phase liquid chromatography (RP-LC). RP-LC would be the primary choice for the analysis of biofluids due to its unpolar stationary phase. HILIC can be complimentary to RP-LC by targeting the resolution of polar metabolites (55).

Metabolomics is the study of metabolite profiling to link cellular pathways to biomolecular mechanisms, and recent advanced in NMR and MS technology will provide further applications in disease diagnosis, altered metabolic pathways in diseases and under drug treatment. Although metabolomics suffers from some limitations, including potential confounding effects (i.e., gender, age, diet, and environment) or limited reproducibility, it has a promising potential in providing both supplementary and complementary data important for biomarker identification and validation (56, 57), as shown, e.g., by Huang et al. (58).

### Interactomics

Genes, proteins, or other biological entities should not be seen in isolation, rather than dynamically interacting in molecular pathways. Such interactions mostly occur on a protein level and, thus, are called PPIs. The so-called interactome is highly dynamic and can vary between different cell and tissue types (59–61), biological or cellular contexts (62, 63), time points, and disease conditions (60, 64, 65). Additionally, proteins can interact with different partners within different pathways based on their function: they can interact on a physical level or within a protein complex (66), through regulation (67), or PTMs (62), which makes it challenging to understand the underlying biology and its molecular mechanistic. For accomplishing this, researchers suggest to systematically map gene and protein interactions (68, 69), and there have already been the effort of linking the interactome to human diseases (65, 70).

### Integration of Heterogeneous Omics Data

As omics data are becoming more easily available for different data types, they can be combined (i.e., integrated) for a better understanding of their relationship and the underlying molecular systems. Data integration has been reported as the combination of data discovery and data exploitation (71), and recent computational advances provide methods for addressing the challenges of integrating heterogeneous data types. These include the identification of network scaffolds by delineating existing interactions between cellular components, the decomposition of such network scaffolds into constituent parts, and the development of system models to simulate and predict the network behavior, expressing as a particular phenotype. Existing studies have been conducted to map cellular pathways on a genome scale to gain insight into cellular responses to environmental perturbations, to develop biomarkers or disease-associated patterns (16). Established methods for analyzing the data include Bayesian Networks (63, 71–73), self-organizing maps (74), or unsupervised network reconstruction (75). A recent review describes how visualization tools can be used to analyze and interpret integrated protein interactions, gene expression, and metabolic profile data (76). Additionally, a recent approach suggested a downstream workflow for integrating heterogeneous data types and procedures for functional analysis that focus on biological pathways by emphasizing the use of curated knowledge resources coupled with expert-guided examination and interpretation of omics data for the selection of potential molecular targets (77).

In the context of CVDs, only few studies have been conducted that use integrated omics data to understand the mechanisms and to identify novel biomarkers. Hou et al. used an omics toolbox with proteomics and transcriptomics to identify novel biomarkers and drug targets in heart failure (78), while Barallobre-Barreiro et al. integrated proteomics and metabolomics to gain mechanistic insights and identify novel biomarkers for CVD (79).

### Network Biology

The analysis of integrated omics data includes the reconstruction, understanding, and modeling of networks that control the behavior of the cell. Summarizing, biological networks are represented as graphs in which components (e.g., genes, proteins, metabolites, reactors, or regulators) are modeled as nodes and their interactions (e.g., regulations, enzymatic reactions, or physical interactions) as edges. These networks can be derived from different types of molecular interactions or on different levels, including PPIs, metabolic interactions, signaling, and transcription–regulatory interactions. In particular, the group of Barabási studied and reviewed the large-scale structure and system-scale function of cellular networks (80–82), as well as the evolutionary mechanisms that might have shaped their development (83).

In systems medicine, we seek to identify the components of complex systems and to model their dynamic interactions (84). Following Lusis and Weiss systems-based approaches involve four logical steps: (a) definition of the system under study (e.g., cardiomyocytes), (b) identification of system components (e.g., proteins regulating a property of interest), (c) determination of component interactions (e.g., network), and (d) modeling of the network dynamics (e.g., how it changes over time or responds to various perturbations) (85). In the context of CVDs, systems-based approaches are becoming more important as the cardiovascular system is highly complex and involves multiple omics data types to be integrated and analyzed. Recent advances in this field could demonstrate the usefulness of reconstructed networks based on integrated omics data. For example, Zhao and Huang reconstructed and analyzed a human heart-specific metabolic network based on transcriptome and proteome data. The tissue specificity of the underlying data was already previously shown to be essential for studying diseases and phenotypes (59, 60), and, based on heart-specific omics data, Zhao and Huang could detect epistatic interactions in the human heart, as well as potential biomarkers for CVD (86). A related study reconstructed a human metabolic network from human cardiomyocytes to accomplish metabolic functions required for maintaining the structural and functional integrity of the cell (87). In contrary, Ryall et al. reconstructed a signaling network specific to cardiac myocyte hypertrophy to identify the most influential species in the cardiac hypertrophy signaling network and demonstrate how different levels of network organization affect myocyte size, transcription factors, and gene expression (88).

### Pathway Analysis

Along with network analysis, a pathway analysis has become a useful method for gaining a detailed insight into biomolecular mechanisms among genes, proteins, metabolites, etc., as it reduces complexity, has increased explanatory power (89) and investigates biological pathways rather than genome-scale networks. Methods and tools for pathway analysis have been developed and studied, such as Pathway-Express, an impact analysis including classical statistics along with crucial factors such as the magnitude of each gene’s expression change, their type and position in the given pathways, and their interactions (90). Other pathway analysis methods and tools include KEGG, KegArray, PathVisio, Ingenuity, and others [for a detailed review, please refer to Wheelock et al. (91)]. These methods integrate different data types and pathway information and perform subsequent pathway analyses, for example, whether certain pathways are enriched for genes differentially expressed under certain conditions (75, 91). These analyses will provide more information on molecular characteristics in the cell under disease conditions or perturbations.

## Methodology – Epidemiological Studies and Experimental Settings

One of the main challenges of systems medicine is the investigation of complex changes and interactions in the human body. In particular, epidemiological studies bear an enormous potential for studying the underlying CVD pathophysiology and evaluating environmental and genetic factors. Population-based and disease cohorts become increasingly available, e.g., the first being the Framingham Heart Study that started in 1948 (92).

### Human Samples

When collecting human samples, their suitability and availability for future applications should be considered. Gene expression and physiology can vary greatly between tissues and cell types, depending on time and function. Secretion samples, including saliva, tear fluid, urine, or feces, are easily accessible but none of them is suitable for all research questions.

In cardiovascular research, blood samples offer a broader range for analyses, such as serum, plasma, or blood cells. Red blood cells are commonly removed by density gradient centrifugation, resulting in the collection of peripheral blood mononuclear cells (PBMCs), including lymphocytes, monocytes, and dendritic cells. Blood cells are often used for functional analyses, e.g., gene expression analyses, while serum and plasma are classical sources for biomarker measurements as they include molecules, such as miRNAs, from cells and tissues from the whole body (93). By contrast, obtaining tissue samples is more challenging, in particular from tissues that are difficult to biopsy (e.g., heart, brain). Especially, cardiac biopsies are extremely invasive and only available when surgery or heart catheter procedures are performed. Major cardiac cell types are fibroblasts, myocytes, endothelial cells, and vascular smooth muscle cells. Additionally, transient cells, such as immune cells, are present in cardiac tissue and, thus, influence gene expression as well (94, 95).

Taken together, epidemiological studies provide the opportunity to study cardiovascular phenotypes over time while obtaining various human samples. Sampling should be carefully considered to take differences between sampling material and possible future applications into account.

### Cell Models

A common way to study molecular functions and interactions is the use of cell cultures. Although living cells can be isolated from fresh biopsies or blood, their life span is limited, which is particularly true for cardiac myocytes (94). Various immortalized or modified cell lines are available, such as THP-1 and HL-1 cells, allowing an easier handling. However, immortalization can change physiology and gene expression of the cells. To study the effect of differential gene expression, various knockdown and overexpression tools are being used, such as RNA interference and transcription activator-like effector nucleases (TALENs). The recently discovered CRISPS/Cas-9 system revolutionizes gene editing by making it faster, easier, available for many species, and allowing multiplexing (96). Additionally, invasive tissue biopsies could be avoided by differentiating induced pluripotent stem cells from skin or PBMCs into cardiac-like cells (97). Collecting these cells during sampling will provide numerous options for future research.

### Animal Models

Animal models are broadly used to study the pathogenesis of complex human diseases. They easily enable standardization of the environment, genetic background, age, and diet. Careful selection of the model has to be done to answer the respective question, taking physiology, biochemistry, and pathophysiology into account. There are various small animal models to study CVD, such as mice, rats, rabbits, and guinea pigs. Mice are by far the most frequently used animal models due to their small size, fast breeding, cost-effectiveness, and the possibility for genetic modifications. However, their cardiovascular system is different from humans (98, 99), as their hearts are smaller, their blood volume is lower, their heart rate is faster, they have no pericardial fat, and a different heart physiology and blood cell composition than humans (99, 100). Most importantly, mice have high antiatherogenic high-density lipoproteins (HDL) and low proatherogenic low-density lipoproteins (LDL), which makes wild-type mice almost resistant to atherosclerosis. To model the pathophysiology of atherosclerosis, mainly two genetically modified mice are being used: ApoE and LDL receptor (Ldlr) knockout mice. ApoE knockout mice lack apolipoprotein E, leading to hypercholesteremia, and spontaneous atherosclerosis development. Even though the lesions resemble human lesions, plasma cholesterol levels are considerably higher and the predominant circulating lipoprotein is VLDL instead of LDL. Additionally, apolipoprotein E influences atherosclerosis development in many ways, e.g., *via* its immunomodulatory properties. Ldlr knockout mice slowly develop atherosclerotic lesions, similar to humans. High-fat diet increases plasma cholesterol levels and leads to a more rapid lesion development. Even though lesion morphology is similar to ApoE knockout mice, Ldlr knockout mice are more similar to the human pathophysiology as hyperlipidemia is milder and plasma lipoprotein profile resembles humans (98). Additionally, various heart failure disease models exist, such as transverse aortic constriction (TAC) and left anterior descending artery-ligation mice (101, 102).

Rats are not as frequently used as mice as they are bigger and genetic modifications were not possible until the introduction of targeted gene disruption using engineered zinc-finger nucleases (ZFNs) in 2009 (103). Similar to mice, wild-type rats do not develop spontaneous atherosclerosis. However, stress can lead to myocardial lesions (100). To date, various genetically modified rat models as well as models for hypertension, MI, and heart failure exist (104–106). Rabbits are well-established models for diet-induced atherosclerosis as their lipoprotein metabolism is similar to humans but they are lacking hepatic lipase. Additionally, there are rabbit models for dyslipidemia, heart failure, and transgenic rabbits (104, 107, 108). Guinea pigs are not so commonly used for the study of CVD, mainly to study arrhythmias and cellular electrophysiology, e.g., with isolated hearts (109, 110). Furthermore, guinea pig models of cardiac hypertrophy and heart failure exist (111). Besides rodents, zebrafish are another comparably new emerging small animal model for CVD. Their short reproductive cycles, easy handling, and the possibility for genetic modifications made them a widely used model for cardiovascular development, regeneration, and recently also arrhythmias (112–114).

Not only small but also large animal models are available for the study of CVD, and they are more similar to human in terms of anatomy, physiology, and size. Pigs have been used as models for CVD for years, particularly to study valvular heart disease and develop surgical procedures. They are similar to human in terms of spontaneous atherosclerotic lesion development, blood composition, heart anatomy, lipid profiles, lipoprotein metabolism, genome, and RNA (99). Besides from pigs, large animal models for CVD include dogs, sheep, goats, and non-human primates (104, 115). However, large animal models are more expensive, need more space and have longer reproductive cycles. Consequently, they are not being used so often and transgenic models, assays, and antibodies are only limited available. Additionally, the number of dogs and non-human primates for cardiovascular research is declining due to ethical aspects, even though their heart physiology is very similar to humans.

## Systems Medicine Approaches in Cardiovascular Disease – Examples

### The *SH2B3* Locus in Relation to Blood Pressure

High blood pressure (BP) is a leading cause of CVD worldwide with a high prevalence in the general population (116). Using large-scale GWASs meta-analyses with up to 200,000 individuals, more than 40 BP SNPs were identified so far. However, those variants explain only 2–3% of BP variation (117), and the molecular mechanisms that lead to increased BP are largely unknown. To identify novel candidate genes involved in BP regulation, Huan et al. (118, 119) applied systems approaches by computationally combining genetic, transcriptomic, and phenotype data. Screening for associations between BP traits and overall gene expression from whole blood of 7,017 individuals (118), 34 distinct genes were significantly related to BP, which in aggregate explained 5–9% of BP variation.

Furthermore, in order to seek for molecular key drivers of BP regulation, Huan et al. (119) and Rotival et al. (120) combined SNP data of known genetic BP variants with gene coexpression networks. In both studies, BP-related variants (121–123) around the gene encoding SH2B adaptor protein 3 (*SH2B3*) were shown to dysregulate highly coexpressed sub-networks. In monocytes, this sub-network contained five BP genes (*CRIP1, RAB11FIP1, MYADM, TIPARP*, and *TREM1*) (120), whereas the sub-network identified in whole blood comprised six trans-regulated BP genes (*ARHGEF40, TAGAP, MYADM, FOS, PPP1R15A*, and *S100A10*) (119). In order to validate their findings from network analysis, transcriptome profiling was performed in whole blood of Sh2b3^−/−^ and wild-type mice (119). A significant overlap of dysregulated genes in networks driven by SH2B3 variants and Sh2b3^−/−^ was shown. Further examination of the role of *SH2B3* in the development of hypertension was performed by Saleh et al., who investigated BP in Sh2b3^−/−^ mice (124) in response to low-dose angiotensin II supplementation. In untreated mice, kidneys of transgenic mice showed greater levels of inflammation, oxidative stress, and glomerular injury in relation to wild-type mice. Such effects were further increased after angiotensin II infusion. In addition, aortas from Sh2b3-deficient mice exhibited stronger inflammation. Bone marrow transplantation of SH2b3^−/−^ into wild-type animals reproduced the hypertensive phenotype, strongly indicating that the predominant effect of SH2B3 on BP is mediated by hematopoietic cells.

### *GUCY* and *CCT7* in Myocardial Infarction

Myocardial infarction is a major cause of death in developed countries, and it is best predicted for middle-aged adults by a positive family history of MI (125), underlining the genetic component of MI. Erdmann et al. (13) recruited a family of 32 members diagnosed with CAD and DNA available in 15 members and performed exome sequencing in 3 affected distant family members and subsequent two-locus linkage analysis. Four rare variants with minor allele frequency below 0.5% were identified and validated in the remaining family members. A loss-of-function mutation in the gene encoding guanylate cyclase 1, soluble, alpha 3 (*GUCY1A3*) and a missense mutation in the chaperonin containing TCP1 subunit 7 (*CCT7*) encoding gene were identified. In subsequent experimental settings, the functional implications of these variants were characterized. Transfection of the *GUCY1A3* mutation into human embryonic kidney (HEK 293) cells and downregulation of CCT7 by siRNA led to a strong reduction of soluble guanylyl cyclase (α1-sGC) α1 levels. Next, the authors investigated α1-sGC levels and NO-dependent cGMP formation in platelets extracted from family members carrying single *GUCY1A3* or *CCT7* mutations, a double mutation, or none of the rare alleles. Carriers of the digenic mutation exhibited a significant reduction of α1-sGC levels and cGMP formation. Since cGMP was known to inhibit platelet activation, representing an important feature of thrombus formation in MI, loss of Gucy1A3 was tested in mice. Gucy1A3-deficient mice showed an increased thrombus formation, indicating an increased risk of MI *via* dysfunctional nitric oxide signaling in rare allele carrying family members.

In summary, both examples used OMICs data to screen for putative disease-causing genes, which were further characterized by *in vitro* experiments or translation into an animal model.

### Cardiac Proteomics and Metabolomics

In a few studies, systems medicine approaches have also been used to assess the interplay between cellular proteins as well as metabolites and oxidative stress in the context of CVD. Mayr et al. combined proteomics and metabolomics approaches to study protein kinase C-mediated cardioprotection by modulation of glucose metabolism (126). A recent study by Chouchani et al. used comparative metabolomics analysis to identify conserved metabolic pathways during ischemia reperfusion, specifically succinate as a metabolic signature of ischemia and subsequently a potential therapeutic target for ischemia–reperfusion injury (127).

## Challenges

**Figure 2:**
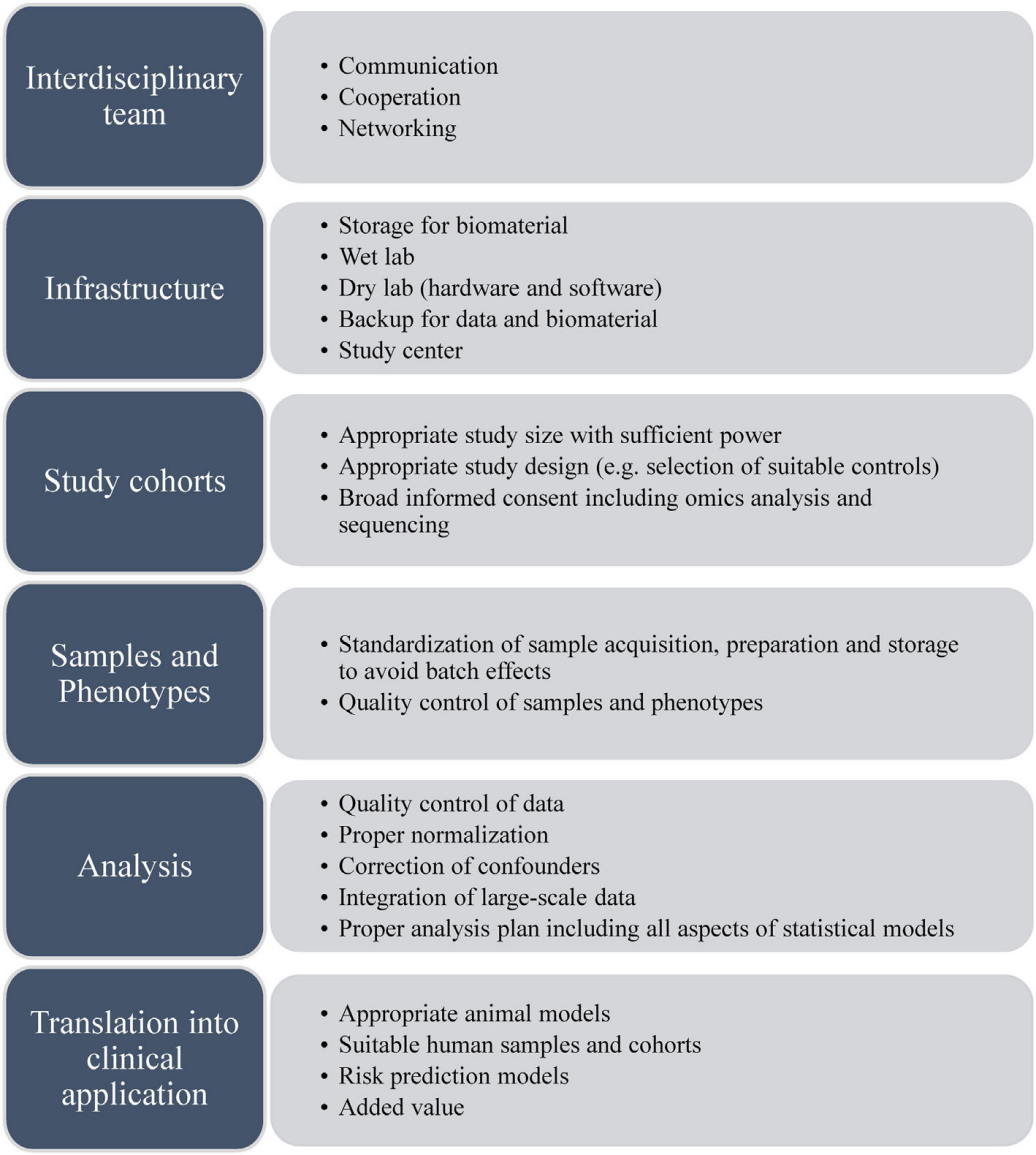
**Challenges for systems medicine approaches**.

As systems medicine is a new, rapidly emerging approach with extensive tools and strategies being developed, researchers are facing multiple challenges. Figure 2 gives an overview about the most prominent challenges to be considered.

## Outlook and Applications

Systems medicine emerged as a powerful tool to study complex diseases by the integration of multidimensional “omics” datasets with data from human studies and experimental laboratory data. CVD is a multifactorial disease and by applying systems medicine approaches, clinical translation might be promoted. Consequently, risk assessment, prevention, and treatment as well as biomarker and drug development can be improved. Due to the interdisciplinary nature, many challenges have to be faced. However, first examples have shown success in the promotion of translational projects in CVD. In a recent study, Kang et al. studied tissue repair based on regenerating tissue from zebrafish fins and cardiac ventricle (128) in order to identify potential novel therapeutic strategies targeting injured human tissue. RNA-Seq from regenerated compared with uninjured tissue revealed strong upregulation of the gene encoding leptin b (lepb), which is paralog to mammalian leptin. ChIP sequencing of histone marks revealed an enhancer element 7 kb upstream of *lepb* (*lepb* linked enhancer LEN), responsible for directing *lepb* gene expression in regenerating tissue, and indicating that LEN can interact with the mammalian transcriptional machinery. Subsequently, in transgenic zebrafish, targeted proliferation of cardiomyocytes could be achieved in resected ventricular apex by inducing the cardiomyocyte mitogen neuregulin 1 (nrg1) *via* LEN fusion to the *nrg1* promoter, showing that transgenic application of LEN was capable to boost injury induced cardiomyocyte proliferation, suggesting novel ways for dynamic therapy upon occurrence of using tissue regeneration enhancer elements.

## Author Contributions

TH, DB, CM, and TZ contributed substantially to the conception, drafting, and revision of the manuscript and approved the final version.

## Conflict of Interest Statement

The authors declare that the research was conducted in the absence of any commercial or financial relationships that could be construed as a potential conflict of interest.
